# Co-Designing Remote Patient Monitoring Technologies for Inpatients: Systematic Review

**DOI:** 10.2196/58144

**Published:** 2024-10-15

**Authors:** Jennifer Sumner, Si Ying Tan, Yuchen Wang, Camille Hui Sze Keck, Eunice Wei Xin Lee, Emily Hwee Hoon Chew, Alexander Wenjun Yip

**Affiliations:** 1 Medical Affairs-Research Alexandra Hospital Singapore Singapore; 2 Alexandra Research Centre for Healthcare In the Virtual Environment (ARCHIVE) Alexandra Hospital Singapore Singapore; 3 Saw Swee Hock School of Public Health National University of Singapore Singapore Singapore; 4 School of Computing National University of Singapore Singapore Singapore; 5 Yong Loo Lin School of Medicine National University of Singapore Singapore Singapore

**Keywords:** remote patient monitoring, technology, inpatient, care transition, systematic review, health technology, patient-centeredness, technology use, effectiveness, study design, assessment, pilot testing, health care, technologies, terminology, quality and consistency, telehealth, telemonitoring

## Abstract

**Background:**

The co-design of health technology enables patient-centeredness and can help reduce barriers to technology use.

**Objective:**

The study objectives were to identify what remote patient monitoring (RPM) technology has been co-designed for inpatients and how effective it is, to identify and describe the co-design approaches used to develop RPM technologies and in which contexts they emerge, and to identify and describe barriers and facilitators of the co-design process.

**Methods:**

We conducted a systematic review of co-designed RPM technologies for inpatients or for the immediate postdischarge period and assessed (1) their effectiveness in improving health outcomes, (2) the co-design approaches used, and (3) barriers and facilitators to the co-design process. Eligible records included those involving stakeholders co-designing RPM technology for use in the inpatient setting or during the immediate postdischarge period. Searches were limited to the English language within the last 10 years. We searched MEDLINE, Embase, CINAHL, PsycInfo, and Science Citation Index (Web of Science) in April 2023. We used the Joanna Briggs Institute critical appraisal checklist for quasi-experimental studies and qualitative research. Findings are presented narratively.

**Results:**

We screened 3334 reports, and 17 projects met the eligibility criteria. Interventions were designed for pre- and postsurgical monitoring (n=6), intensive care monitoring (n=2), posttransplant monitoring (n=3), rehabilitation (n=4), acute inpatients (n=1), and postpartum care (n=1). No projects evaluated the efficacy of their co-designed RPM technology. Three pilot studies reported clinical outcomes; their risk of bias was low to moderate. Pilot evaluations (11/17) also focused on nonclinical outcomes such as usability, usefulness, feasibility, and satisfaction. Common co-design approaches included needs assessment or ideation (16/17), prototyping (15/17), and pilot testing (11/17). The most commonly reported challenge to the co-design process was the generalizability of findings, closely followed by time and resource constraints and participant bias. Stakeholders’ perceived value was the most frequently reported enabler of co-design. Other enablers included continued stakeholder engagement and methodological factors (ie, the use of flexible mixed method approaches and prototyping).

**Conclusions:**

Co-design methods can help enhance interventions’ relevance, usability, and adoption. While included studies measured usability, satisfaction, and acceptability—critical factors for successful implementation and uptake—we could not determine the clinical effectiveness of co-designed RPM technologies. A stronger commitment to clinical evaluation is needed. Studies’ use of diverse co-design approaches can foster stakeholder inclusivity, but greater standardization in co-design terminology is needed to improve the quality and consistency of co-design research.

## Introduction

In recent decades, health care systems have significantly transformed thanks to innovations in wearable devices, telemedicine, and artificial intelligence [1,2]. Adopting technology into health care brings various advantages, including enhancements to patient-centric care, gains in operational efficiency, and diversification of care delivery approaches. Additionally, care providers are increasingly viewing technology as a solution to workforce shortages [3,4]. One area undergoing rapid growth is remote patient monitoring (RPM) [5-7]. In brief, RPM refers to tools that capture health data that can be reviewed remotely [8]. RPM technologies encompass a wide range of tools, including wearable devices, telehealth platforms, mobile apps, implantable devices, and Internet of Things devices.

RPM technologies serve diverse purposes, from treating and observing acute conditions in the inpatient and care transition period to longer-term maintenance of chronic diseases in the outpatient setting. During inpatient care, RPM can enable continuous, real-time monitoring of patients, facilitating early detection of complications, and allowing for timely interventions [5,6,9-12]. As patients transition from inpatient to care in their homes, RPM ensures continuity in monitoring, supporting adherence to discharge instructions, and facilitating prompt responses to declines in health [5,6,9-12]. Furthermore, RPM technologies can assist with earlier discharge or hospital-at-home care models, reducing the length of stay [5,6,9-12]. In the outpatient setting, RPM technologies shift away from acute care to long-term maintenance of chronic diseases, providing oversight and self-management support [5,6,9-12].

While RPM technology holds significant promise, many potentially impactful technologies fail to be adopted or scaled in practice [13]. One prevailing theory suggests that inadequate stakeholder engagement during the technology development phase inhibits the relevance and adoption of the resulting device [13,14]. The omission of stakeholder insights can lead to poor consideration of user needs, leading to the creation of solutions that fail to fulfill their intended purpose effectively. Accordingly, participatory design of technology is advisable to enhance effectiveness and acceptance [15]; one such approach is co-design. Co-design helps to ensure that solutions closely align with user requirements, improving the relevance and usability of the final product and broader adoption [15,16].

Co-design involves a collaborative effort among stakeholders, including technologists, patients, and health care providers, to develop interventions or services [15,17,18]. The process is iterative and entails multiple phases of development, refinement, and evaluation to achieve the final outcome. Built on the premise that users are experts in their experiences, co-design engages users to improve and innovate services or products [19,20]. There are multiple successful examples of co-designed technology in health care that have improved patient satisfaction, enhanced outcomes, and reduced costs [17,21-24].

As investment and the need for RPM technology grows [25], optimizing the technology design becomes crucial to meet stakeholders’ needs and ensure uptake. Therefore, our aim was to gain a comprehensive understanding of the role and impact of participatory design methods, specifically co-design, in the RPM domain. As RPM technology in the inpatient and outpatient settings has unique purposes and requirements, we specifically focus on RPM technology during the inpatient and care transition period to home (ie, RPM technology for acute rather than chronic care needs). Our specific objectives were to identify what RPM technology has been co-designed for inpatients and how effective it is, to identify and describe the co-design approaches used to develop RPM technologies and in which contexts they emerge, and to identify and describe barriers and facilitators of the co-design process.

## Methods

### Study Design

This study is reported and conducted according to the PRISMA (Preferred Reporting Items for Systematic Reviews and Meta-Analyses) guidelines [26]. The systematic review protocol was prospectively registered on the PROSPERO database of systematic reviews (registration CRD42024505427). A copy of the PRISMA checklist is included in Multimedia Appendix 1.

### Literature Search

We searched MEDLINE, Embase, CINAHL, PsycInfo, and Science Citation Index (Web of Science) in April 2023 using a combination of MeSH terms and keywords around the themes of co-design and RPM technology. The initial strategy was developed in MEDLINE and adapted for use in the other databases. The reference lists of included papers were also searched for further relevant references. A copy of the MEDLINE search strategy is included in Multimedia Appendix 2.

### Study Selection

Citations were downloaded and managed in EndNote X9 (Clarivate). Five reviewers (JS, YW, CHSK, EWXL, and EHHC) independently screened titles and abstracts for inclusion according to predefined screening criteria (Textbox 1). When reviewers could not reach a screening decision, the group discussed the paper until a consensus was reached. All reviewers screened the first 50 papers and then met to discuss screening alignment and disagreements; this helped to ensure screening consistency among the reviewers and aided with the refinement of the eligibility criteria. Shortlisted papers were then full-text screened by 4 reviewers in pairs (YW, CHSK, EWXL, and EHHC). Uncertainties around eligibility were discussed and resolved by a fifth reviewer (JS or SYT). If publications were unclear, efforts were made to contact the authors and obtain further details.

**Participants, intervention, control, outcomes, and study types eligibility criteria.**

Participants: Any stakeholders involved in the use or development of remote patient monitoring (RPM) technologies, including health care workers, patients admitted to hospital, technologists, designers, or researchers.

Intervention: Any co-designed RPM technology for use in the inpatient hospital setting or during the immediate postdischarge period (ie, care transition). RPM technology may include but is not limited to wearable devices, telehealth platforms, mobile apps, implantable devices, and Internet of Things devices. We defined co-design as “The participation and equal collaboration between service providers, users, carers and the broader community to co-design health-related technology.” To be considered as a “co-design” study, studies had to include the following attributes: (1) involvement of all relevant stakeholders; AND (2) evidence of collaboration between stakeholders beyond only information gathering from consumers; AND (3) evidence that the stakeholders were involved in the development process at more than 1 time point, that is, demonstrates meaningful contribution of stakeholders to project; AND (4) multiple, iterative stages of development, such as needs assessment, ideation, prototyping, pilot testing (ie, usability), and impact evaluation.

Control: For interventional studies, the control group is defined as those not using a co-designed technology. Studies without a control group are also eligible for inclusion if the other criteria are met.

Outcomes: Any papers with clinical or patient-reported health and well-being outcomes. Papers with data on the experience of the co-design process, including facilitators of and barriers to co-design, were also eligible.

Study types: All study types were considered: that is, experimental, observational, quantitative, and qualitative.

Other: Studies were restricted to English language-only papers, and the search was limited to the last 10 years. The date restriction was chosen to identify relevant technologies for the modern-day context.

### Data Extraction and Management

Five reviewers were involved in data extraction (JS, YW, CHSK, EWXL, and EHHC). One reviewer extracted the data, and a different reviewer checked the data for accuracy. Any disagreements were discussed and resolved by a third reviewer. Extracted data items included study and population characteristics, intervention details, co-design process information, facilitators and barriers to the co-design process, and outcome measures. In cases where multiple publications were identified for one study, data from the primary study publication were extracted, and the additional publications were scanned for additional information. The extraction sheet was piloted on 3 papers, and refinements were made to the extraction sheet before the remaining papers were extracted.

### Quality Assessment

We used the Joanna Briggs Institute critical appraisal checklist to assess the risk of bias for quasi-experimental studies that evaluated a final intervention. The checklist includes 9 questions grouped into 7 domains, which prompt the reviewer to assess the risk of bias [27]. For qualitative studies that evaluated a final intervention, we used the Joanna Briggs Institute critical appraisal for qualitative research [28]. The checklist contains 10 items, which prompt the reviewer to appraise the quality of the study. One reviewer assessed each study (JS), and a second reviewer checked the decisions (YW). Any disagreements were discussed until a consensus was reached.

### Data Synthesis

Study outcomes are presented in a narrative synthesis. We descriptively report on the study characteristics, type and purpose of the included interventions, the target population for each intervention, and the technology components used. Each intervention was categorized into 1 of 6 groups based on its primary purpose: educational, monitoring of health, adherence, safety alerts, 2-way communication, and intervention delivery. Categories were agreed upon through discussion. We then plotted the categories (inner circle) into a sunburst plot with associated examples (outer circle). As a meta-analysis was impossible due to a lack of data, we summarize the number of studies reporting efficacy measures, the types of outcomes measured, and the corresponding impact as text. Co-design approaches are described narratively, highlighting the typical stages of co-design undertaken, the methods used, those involved in the process, and the evaluation metrics reported. Finally, we describe the barriers and enablers of the co-design process narratively. In addition, we generated a word cloud to visualize the co-design process barriers and enablers and their frequency of occurrence. To generate the word cloud, the study team first categorized the types of barriers and enablers to co-design through mutual discussion. One reviewer (JS) applied the categories to the data, and a second reviewer then cross-checked the categories for relevance and accuracy (SYT). The number of publications mentioning a particular barrier or enabler was then mapped to each category. A web-based word cloud generator, Flourish, was then used to create the image.

## Results

### Overview

**Figure 1:**
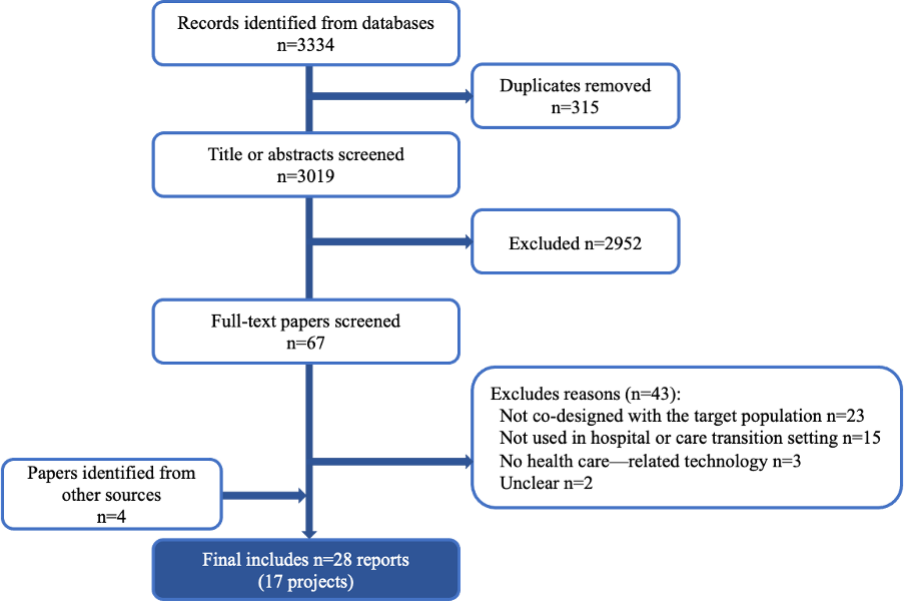
PRISMA (Preferred Reporting Items for Systematic Reviews and Meta-Analyses) flow diagram.

We identified 3334 reports, including 315 duplicates, which we removed. Independent screening of the remaining 3019 reports led to a further exclusion of 2952 reports. Following the full-text screening, we excluded 39 reports, leaving 28 included (for 17 projects; Figure 1). The most common reason for exclusion was due to the use of non–co-design methodologies to develop the RPM technology. For example, some studies only engaged in needs assessments and did not actively collaborate with stakeholders. In other cases, the design team did not include key stakeholders (ie, direct technology users). A few studies reported the start of a co-design process but had yet to undertake the work; these we excluded. Finally, RPM technologies not developed for the hospital setting or the immediate postdischarge period (ie, care transition) were excluded. For example, we excluded community-based self-management tools designed for long-term use.

### Study Characteristics

Table 1 presents the characteristics of the 17 included projects. Projects were from Europe (n=10) [29-38], the United States (n=4) [39-42], Canada (n=1) [43], New Zealand (n=1) [44], and Sri Lanka (n=1) [45]. Interventions were designed for postsurgical monitoring (n=5) [29-32,39], patients in intensive care (n=2) [33,45], posttransplant monitoring (n=3) [34,35,40], rehabilitation (n=4) [36-38,43], acute inpatients (n=1) [44], presurgical monitoring (n=1) [41], and postpartum care (n=1) [42].

**Figure 2:**
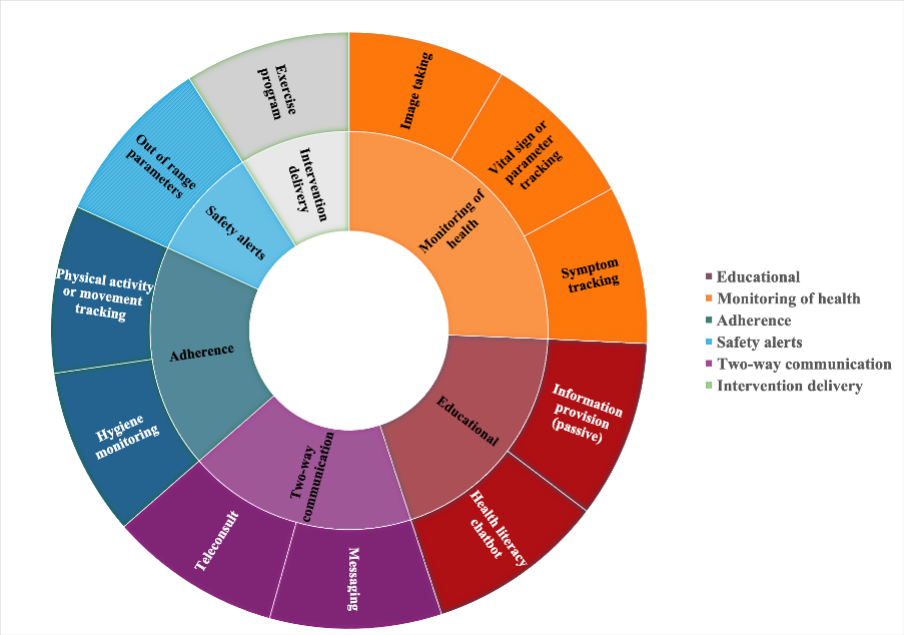
Sunburst plot of main technology purpose (inner circle) and associated examples from included reports (outer circle).

Figure 2 shows the 6 categories of interventions (based on their primary purpose: educational, monitoring of health, adherence, safety alerts, 2-way communication, and intervention delivery) and specific examples from the included reports. Interventions used different technology components including apps (15/17) [30-32,34-45], wearable sensors (3/17) [32,33,36], dashboards (3/17) [32-34], a chatbot (1/17) [30], a video consultation platform (1/17) [34], a smart garment (1/17) [36], and a website (1/17) [29].

**Table 1:** Included study characteristics.

Author (year), country	Target population and health technology developed	Stages of co-design conducted	Stakeholders involved
Hartup et al (2022, 2023) [29,46], Robinson et al (2023) [47], United Kingdom	Patients undergoing surgery (breast). A web-based intervention (ePainQ) to monitor postoperative pain and facilitate advice provision.	Needs assessment n=N/R^a^ Pilot n=69	Patients, health care professionals, and academics
Londral et al (2022) [30], Portugal	Patients undergoing surgery (cardiac). A postoperative digital telemonitoring service to monitor postsurgical wounds and improve health literacy.	Needs assessment n=N/R Ideation n=N/R Prototyping n=30 Pilot n=60	Patients, family caregivers, surgeons, nurses, and technologists
Miller et al (2020) [31], United Kingdom	Patients undergoing surgery (colorectal). An app to support self-care and monitoring post-surgery.	Needs assessment n=19 Ideation n>19 Prototyping n=30	Patients, physicians, nurses, and pharmacy staff
Naeemabadi et al (2019, 2020) [32,48], Denmark	Patients undergoing surgery (knee replacement). A telerehabilitation program solution using sensor technologies to facilitate in-home rehabilitation.	Needs assessment n=8 Ideation n=19 Prototyping n=18 Pilot 1 n=7; pilot 2 n=4	Patients, health care professionals (physiotherapists, nurses, and orthopedic surgeons), researchers, students, and software developers
Sanger et al (2014, 2016) [39,49], Gunter et al (2016, 2018) [50,51], United States	Patients with surgical wounds. A mobile health wound monitoring app: Mobile Post-Operative Wound Evaluator.	Needs assessment n=37 Ideation n=24 Prototyping n=21 Pilot 1 n=12; pilot 2 n=40	Patients, providers, health informatics specialists, interaction designers, computer scientists, and patient advisors
Kariyawasam et al (2017) [45], Sri Lanka	Patients in the surgical intensive care unit. An app to support monitoring of hand hygiene compliance.	Needs assessment n=N/R Ideation n=N/R Prototyping n=N/R Pilot n=N/R	Intensive care unit staff, hospital infection control staff, hospital administrators, and microbiologists
Poncette et al (2022) [33], Germany	Patients in intensive care. Redesign of the user interface for the Vital Sync (version 2.4; Medtronic); a web-based inpatient monitoring platform.	Needs assessment n=10 Ideation n=2 Prototyping n=10	Physicians and medical students
Vaughn et al (2020) [40], United States	Patients undergoing blood and marrow transplant. An app for posttransplant symptom monitoring.	Prototyping n=32 Pilot n=36	Patients, a physician researcher, nurse scientists, a nurse practitioner, a pediatric nurse, and child therapists
Duettmann et al (2021) [34], Germany	Patients undergoing a kidney transplant. An app to monitor posttransplant vital signs, well-being, and medication intake and facilitate physician communications and consultations.	Needs assessment n=N/R Ideation n=N/R Prototyping n=N/R Pilot n=131	Patients and health care professionals
Nielsen et al (2019, 2020) [35,52-54], Denmark	Patients undergoing a kidney transplant. An app to replace in-person follows through tracking of vital sign data, other self-reported health measures, medication use, and a messaging function.	Needs assessment n=48 Ideation n=44 Prototyping n=19 Pilot 1 n=36	Patients, patient representatives, doctors, nurses, nursing assistants, secretaries, physiotherapists, dieticians, IT designers, innovation consultants, and researchers
Baig et al (2014, 2015, 2020) [44,55,56], New Zealand	Patients in acute care (general medicine). An app that interprets data on blood pressure, heart rate, oxygen saturation, blood glucose, and temperature for patient monitoring in acute care settings.	Needs assessment N/A^b^ Prototyping n=10 Pilot 1 n=20; pilot 2 n=30	Nurses, rapid response team members, or emergency response team members
Blair et al (2022) [41], United States	Patients with a congenital heart defect. An app to support home monitoring during the high-risk interstage period (period between surgical procedures) after discharge.	Needs assessment n=6 (review of web-based blogs) Ideation n=N/R Pilot n=11	Caregivers and clinicians
Logsdon et al (2020) [42], United States	Postpartum women. An app to increase a mother’s ability to monitor her own health after childbirth.	Ideation n=5 Prototyping 1 n=5; prototyping 2 n=22	Postpartum women
Mortenson et al (2019) [43], Canada	Patients with spinal cord injury (rehabilitation). An app to build self-management skills needed to prevent secondary complications following recent inpatient rehabilitation for spinal cord injury.	Needs assessment n=N/R Ideation n=N/R Prototyping n=N/R	Patients with spinal cord injury and formal and informal caregivers
Perego et al (2022) [36], Italy	Patients who experienced a stroke (rehabilitation). A wearable system for monitoring and evaluating motor rehabilitation activities at home.	Needs assessment n=N/R Ideation n=N/R Prototyping n=10	Patients, physicians and other health care workers from rehabilitation, technologists, and designers
Timmerman et al (2016) [37], Netherlands	Patients with lung cancer (rehabilitation). An app-based rehabilitation program, including physical exercise and symptom or physical activity tracking.	Needs assessment n=21 Ideation n=10 Prototyping n=17	Patients, surgeons, a rehabilitation physician, physiotherapists, and a nurse
An et al (2021) [38], United Kingdom	Patients with chronic obstructive pulmonary disease (rehabilitation). A digital rehabilitation app following hospitalization for chronic obstructive pulmonary disease exacerbation.	Needs assessment n=26 Ideation n=26 Prototyping n=26 Pilot 1 n=7	Patients, providers, and designers

^a^N/R: not reported.

^b^N/A: not applicable.

### Technology Effectiveness

None of the 17 projects included evaluated the efficacy of their co-designed RPM technology. Three pilot studies [30,45,51] reported on clinical outcomes. Londral et al [30] compared a prospective sample of patients (n=29) using a postoperative digital telemonitoring service to a retrospectively matched control group receiving usual care (n=30). They found that the average critical incident rate (readmission, surgery, or death) was lower in the telemonitored group compared to the control group (n=60; *P*=.01). Kariyawasam et al [45] conducted an uncontrolled pilot (sample size unknown) assessing the effectiveness of a hand hygiene monitoring platform. They found the proportion of participants with positive cultures after an intensive care unit stay, antibiotic use after an intensive care unit stay, and signs and symptoms of infection were 12%, 79%, and 60%, respectively. Gunter et al [51] conducted an uncontrolled pilot (n=40) evaluating a mobile wound monitoring app. They identified 7 wound complications and 1 false negative during the 2-week evaluation period. Two participants were also readmitted to the hospital but for reasons unrelated to wound infection.

### Quality Assessment

For the 3 pilot studies that reported clinical outcomes, the risk of bias was low to moderate across the domains. Londral et al [30] was found to have a moderate risk of bias in 5 domains, Kariyawasam et al [45] in 2 domains, and Gunter et al [51] in 3 domains. All other domains had a low risk of bias. For the remaining quantitative studies (not reporting clinical outcomes), a moderate risk of bias was reported in 1 domain for each study [29,34,44,50,56] and an unclear risk of bias was reported in 5 domains for one study [34] and in 2 domains for another [29]. All other domains had a low risk of bias. For the studies reporting qualitative outcomes, bias was detected in 3 domains for 2 studies [40,48] and in 2 domains for 3 studies [38,41,48]. Unclear bias was also found in 2 domains for one study [38] and in 1 domain for another study [40]. All other domains had a low risk of bias. The quality assessment findings are summarized in Multimedia Appendices 3 and 4.

### Co-Design Approaches Used

#### Overview

Co-design is an iterative process typically involving several stages. We found that not every study conducted every co-design stage (Table 1). In total, 12 of the 17 projects included needs assessment, ideation with stakeholders, and solution prototyping [30-39,43,45]. Two studies did not report on a needs assessment phase [40,42], 1 of which went straight to prototyping [40]. Three studies did not report on ideation activities [29,40,44], and 2 studies developed no prototype and went straight to perform pilot evaluations [29,41].

Health care providers were the most commonly included stakeholders in co-design (16/17) [29-41,43-45]. All but 3 studies involved patients or caregivers; 2 were for vital sign tracking systems [33,44], and 1 was for monitoring staff hygiene [45]. The lack of patient involvement was unsurprising, as patients were not users in these examples. Finally, technologists or designers were involved in 6 of 17 studies [30,32,35,36,38,39]. Other less common stakeholders were health service administrators and patient advocacy groups. No study involved commissioners, service managers, or policy makers.

#### Needs and Ideation

The number of participants involved in this co-design stage ranged between 1 and 48. Methodological approaches common to both stages were interviews, focus groups, literature reviews, workshops, and questionnaires. Other approaches (only used in needs assessment) included ethnographic observations, analysis of blog data, and audit of existing services. The method only used in ideation was written feedback from stakeholders or domain experts (ie, email correspondence).

#### Prototyping

Prototyping involved the creation of mock-ups of the proposed intervention which stakeholders fed back on. The number of participants involved in these activities ranged from 10 to 48, and in some cases, multiple iterations of the prototype were developed and refined with stakeholders. Prototypes ranged in sophistication from light fidelity pen and paper sketches (eg, storyboard) to fully functional interventions (eg, fully functional apps). Stakeholder feedback on the prototype was sought through interviews, focus groups, work groups, structured prototype interaction with a task list, unguided prototype interaction, think-aloud feedback, and questionnaires.

#### Pilot Evaluation

Pilot studies typically assessed nonclinical outcomes such as usability, usefulness, feasibility, and satisfaction (Table 2). Clinical outcomes were reported in 3 projects [30,45,51]. Studies ranged in size from 7 to 131 participants. Participant feedback was gathered through interviews, focus groups, questionnaires, and utility metrics.

**Table 2:** Pilot study evaluation metrics.

Study	Outcomes	Measurement approach
Baig et al (2014, 2015, 2020) [44,55,56]	Satisfaction, mobility, usability, comfort to operate, and acceptability	Questionnaire
Blair et al (2022) [41]	Usability and acceptability	Interviews
Duettmann et al (2021) [34]	Uptake	Enrollment figures
Hartup et al (2022, 2023) [29,46], Robinson et al (2023) [47]	Acceptability, usability, and usefulness	Questionnaires (EORTC C30 and BR23^a^, EQ-5D, HADS^b^, BPI^c^, and patient activation) Interviews
Kariyawasam et al (2017) [45]	App use, behavioral attitudinal changes, user experience, percentage with positive cultures, percentage with antibiotic use after intensive care unit stay, and signs and symptoms of infection	App usage metrics Interviews or focus groups Observations Clinical data
Londral et al (2022) [30]	User experience, patient adoption, recovery experience, adherence, requirement for technology support, and critical incident rate	User experience questionnaire Net promoter score Questionnaire with open-ended questions App usage metrics Clinical data
Naeemabadi et al (2019, 2020) [32,48]	User experience, satisfaction, and usability	Observations Interviews Questionnaire
Nielsen et al (2019, 2020) [35,52-54]	User experience	Interviews or focus groups
Sanger et al (2014, 2016) [39,49], Gunter et al (2016, 2018) [50,51]	Usability, satisfaction, adherence, satisfaction, burden to workflows, surgical site infection, and hospital readmission	Systems usability questionnaire Interviews (staff and patients) App usage metrics Clinical data
Vaughn et al (2020) [40]	User experience, feasibility, usability, and acceptability	Interviews Questionnaire
An et al (2021) [38]	Usability, usefulness, satisfaction, completion of tasks, and error rate	Interviews Completion of tasks and task error rate

^a^EORTC C30 BR23: European Organisation for the Research and Treatment of Cancer Quality of Life questionnaire and Breast Cancer Specific Module.

^b^HADS: Hospital and Anxiety Depression Scale.

^c^BPI: Brief Pain Inventory.

### Barriers and Facilitators of the Co-Design Process

In total, 16 projects reported on the barriers and facilitators of the co-design process (Multimedia Appendix 5) [30-45]. The most common barrier to co-design is related to the representativeness of the design group and, correspondingly, the generalizability of the output [30,32,33,35,39,40,43]. Studies noted that co-design groups were often small and may not represent the views of a wider audience. Another commonly reported barrier to co-design was bias [30,37,39,43]. Some mentioned that the methodological approach may have influenced the project direction and biased the output. Others reflected that stakeholder feedback may have been biased due to a predilection for socially desirable responses and the involvement of “early adopters” who may not represent others’ views. The time- and resource-consuming nature of co-design was also frequently noted and impacted studies in several ways [30-32,35,43], for instance, the inability to create comprehensive prototypes for participant feedback, difficulties maintaining user engagement with the project, and constraints on the number of participants who could be involved in the co-design process. Other less common barriers included stakeholder conflict and the requirement for good facilitation, stakeholder recruitment challenges, logistical challenges (ie, scheduling), and lack of institutional buy-in, which may impact adoption [31-33,35,39].

Perceived value or buy-in was the most commonly reported enabler of co-design [30,33-35,37]. Buy-in helped maintain stakeholder engagement over time and will ultimately aid with adoption. Furthermore, sustained stakeholder engagement ensured continuous feedback as concepts developed, the ability to effectively troubleshoot, and the enablement of human-centered design [30,31,33,34,41]. Flexibility in the methods was also noted; this helped to deal with logistical barriers and accommodate stakeholders’ needs [31,35]. For instance, stakeholders may have full-time jobs and are unable to participate during “office hours.” Other less common enablers included the importance of close communication, the use of prototyping that aided early usability assessments, mutual learning among stakeholders, early stakeholder engagement, participant ownership, and the use of mixed methodologies [30,34-36,39].

## Discussion

### Principal Findings

In this systematic review of 17 co-designed RPM technologies for the hospital setting or during the immediate postdischarge period, we found no studies evaluating the efficacy of their devices. In the included papers, the co-design development phases spanned needs assessment, ideation, prototyping, and pilot testing (ie, focus on usability). The most commonly reported challenges to the co-design process were generalizability of the output, time and resource constraints, and participant bias. Common enablers included ongoing engagement and stakeholder buy-in. Overall, authors frequently assess usability, satisfaction, and acceptability, facilitating the development of stakeholder-centric solutions. Nevertheless, no reported data supported the clinical effectiveness of the co-designed interventions.

Existing evidence from non–co-designed RPM technologies demonstrates promising results in enhancing patient safety, reducing the length of stay while maintaining adherence [9-12], and proving to be cost-effective [57,58]. However, we found no projects evaluating the clinical impact of co-designed RPM technologies. This evidence gap is consistent with other reviews of co-designed health care interventions in different settings, which consistently highlight a lack of clinical evaluation for co-designed interventions [24,59,60]. While metrics such as usability, satisfaction, and acceptability are frequently measured and are certainly important for successful implementation and adoption [61], the absence of robust clinical evidence remains a critical concern that warrants attention.

Notwithstanding the need for technological innovation to improve health care and address ongoing sustainability challenges [62], health systems must be mindful of introducing new vulnerabilities through the introduction of technology. Populations lacking digital literacy or access to technology, such as older patients, individuals with disabilities, or those with lower socioeconomic status, may struggle to use and adhere to RPM approaches, exacerbating existing health disparities [63,64]. Accordingly, involving these vulnerable populations in the design process becomes all the more crucial. Stakeholders can offer invaluable insights into their unique needs, preferences, and challenges, improving an intervention’s usability, effectiveness, and safety [15]. Moreover, stakeholder involvement can help to mitigate barriers to adoption [13]. While our included studies targeted a range of conditions and scenarios, there was no specific focus on vulnerable populations. Future efforts should consider the active engagement of these demographics to ensure technology relevance and usability.

To engage stakeholders effectively, including vulnerable groups, the co-design literature emphasizes flexibility in the approach [65-67], a principle reflected in our included studies. We found a wide variety in terms of the stages of co-design undertaken (ie, needs, ideation, and prototyping), the number of stakeholders involved, and the methods used. On the one hand, the diversity in the co-design process helps accommodate the unique needs and abilities of each stakeholder and aids in developing equitable solutions. For example, working professionals are typically time-constrained, while patients with underlying conditions may struggle to engage in conventional discussions. On the other hand, diversity in co-design processes may also be attributed to inconsistencies in the co-design terminology and definition [60]. Accordingly, these different interpretations of the concept of co-design may influence how researchers execute their study, making it difficult to compare and evaluate the value of co-design outcomes across different projects. In time, the development of the Cocreation Research Standards (CORES) may help with greater standardization [68].

### Recommendations

While RPM technologies have several reported benefits, avoiding exacerbation of existing health disparities through the introduction of technology is crucial. Designers may wish to consider frameworks, such as the digital health equity framework [63], to inform their co-design process. Furthermore, those engaging in co-design of technology must be mindful to accommodate mixed stakeholder capabilities. To ensure inclusivity and representativeness, researchers should strive to involve a wide range of individuals and groups. However, this will require careful management of stakeholder interactions. For instance, ensuring a shared understanding of the problem and technological possibilities will help to aid stakeholder engagement. Using a variety of engagement approaches will also give different stakeholders a platform to be involved and prevent unwanted power dynamics that may hinder collaboration. Finally, greater investment in the impact evaluation of co-designed technologies is required to establish the clinical value of co-designed interventions and further justify the resource-intensive process.

### Limitations

Due to variability in the co-design terminology, it is feasible that we missed some relevant papers. We attempted to minimize this risk by incorporating a broad range of terms in our search strategy and working with an information specialist. As no unified definition for co-design exists, we developed our own criteria, others may have different interpretations. Thus, we may have excluded relevant papers. We also limited our search to the past 10 years, and we felt this was necessary due to rapid development within the RPM field.

### Conclusions

Health care organizations increasingly adopt co-design to improve intervention relevance, usability, and subsequent adoption. However, we could not conclude the effectiveness of co-designed RPM technologies. A more significant commitment to clinical evaluation is needed. Greater standardization in the co-design terminology is also needed to improve the quality and consistency of co-design research.

## Appendix

Multimedia Appendix 1 PRISMA (Preferred Reporting Items for Systematic Reviews and Meta-Analyses) checklist.

Multimedia Appendix 2 Search strategy.

Multimedia Appendix 3 Results of quality assessment for experimental studies.

Multimedia Appendix 4 Results of quality assessment for qualitative studies.

Multimedia Appendix 5 Word cloud depicting frequency of reported barriers (dark blue) and enablers (light blue) to the co-design process (larger words represent higher frequency of reporting).

## Abbreviations

CORES: Cocreation Research Standards
PRISMA: Preferred Reporting Items for Systematic Reviews and Meta-Analyses
RPM: remote patient monitoring
