# Supplementary material for: Co-Designing Remote Patient Monitoring Technologies for Inpatients: Systematic Review
Source: J Med Internet Res. 2024 Oct 15;26:e58144. doi: 10.2196/58144 (PMC11522647; doi:10.2196/58144)
Supplement: Multimedia Appendix 2 [file jmir_v26i1e58144_app2.pdf]

MEDLINE Search Strategy

((("biosensing techniques"[MeSH Terms] OR "Remote sensing technology"[MeSH] OR "remote sensing"[text word] OR "On body sensor"[text word] OR Biosensor*[text word] OR "Wearable device"[text word] OR "Constant health monitoring"[text word] OR "Wireless technology"[text word] OR "wearable sensor"[text word] OR "wearable"[text word] OR "medical sensor"[text word] OR "Body Sensor"[text word] OR "Passive monitor"[text word] OR "wireless monitor"[text word] OR "monitoring device"[text word] OR "wireless sensor"[text word])) OR (("Remote monitoring"[text word] OR "Remote patient monitoring"[text word] OR "self-monitoring"[text word] OR "self tracking"[text word] OR "remote tracking"[text word] OR "home monitoring"[text word] OR "wireless monitoring"[text word] OR "online monitoring"[text word] OR "online tracking"[text word] OR "telemonitoring"[text word] OR "ambulatory monitoring"[text word]) AND ("e-health"[text word] OR "m-health"[text word] OR "mobile"[text word] OR "mobile health"[text word] OR "telehealth"[text word] OR "telemedicine"[text word] OR "teleICU"[text word] OR "tele-ICU" [text word] OR "hospital at home"[text word] OR "digital health"[text word] OR "digital medicine"[text word] OR (("smartphone"[MeSH Terms] OR "smartphone"[All Fields]) AND text[All Fields] AND word[All Fields]) OR "social network"[text word] OR "Web based"[text word] OR "online portal"[text word] OR "internet based"[text word] OR "cell phone"[text word] OR "mobile phone"[text word])) NOT ("self-monitoring"[text word] OR "self-management"[text word]))

AND

("Patient and public involvement"[Text Word] OR (Patient participation[Text Word]) OR (Collective creativity[Text Word]) OR (Empathetic* adj2 design*[Text Word]) OR (Value sensitive design*[Text Word]) OR (Human cent* design*[Text Word]) OR (Inclusive design*[Text Word]) OR (Action adj2 research) OR (Lived experience[Text Word]) OR (Open innovation[Text Word]) OR (Collective intelligence[Text Word]) OR (Community adj1 participa*[Text Word]) OR (Consumer adj (drive[Text Word] OR driven[Text Word])) OR (Experience based design*[Text Word]) OR (Participa* ergonomics[Text Word]) OR (Participa* design*[Text Word]) OR (Persona based design*[Text Word]) OR (Co-produce[Text Word] OR co-producing[Text Word] OR co-production[Text Word] OR Coproduc*[Text Word]) OR (Co-creat*[Text Word] OR Cocreat*[Text Word]) OR (Co-design*[Text Word] OR Codesign*[Text Word]) OR (Co-care[Text Word] OR co-caring[Text Word] OR co-cared[Text Word] OR Cocar*[Text Word]) OR (Co-commiss*[Text Word] OR Cocommiss*[Text Word]) OR (Co-decide[Text Word] OR co-decision[Text Word] OR co-decided[Text Word] OR Codeci*[Text Word]) OR (Co-deliver*[Text Word] OR Codeliver*[Text Word]) OR (Co-evaluat*[Text Word] OR Coevaluat*[Text Word]) OR (Co-implement*[Text Word] OR Coimplement*[Text Word]) OR (Co-construct*[Text Word] OR Coconstruct*[Text Word]) OR (Co-innovat*[Text Word] OR Coinnovat*[Text Word]) OR (Co-learn*[Text Word] OR Colearn*[Text Word]))
