# Supplementary material for: Co-Designing Remote Patient Monitoring Technologies for Inpatients: Systematic Review
Source: J Med Internet Res. 2024 Oct 15;26:e58144. doi: 10.2196/58144 (PMC11522647; doi:10.2196/58144)
Supplement: Multimedia Appendix 3 [file jmir_v26i1e58144_app3.pdf]

Study ID	Domain								
	**1. Temporal precedence**	**2. Selection and Allocation**	**3. Confounding factors**	**4. Administration of the intervention**	**5. Measurement pre-post**	**6. Measurement consistency**	**7. Measurement reliability**	**8. Participant retention**	**9. Statistical conclusion and validity**
Londral et al (2022) [30]	Yes	Yes- historic control	Unclear- demographics for both groups not reported	Unclear- historic control	N/A- event rates	Unclear-historic control	Unclear- historic control	No- impact of drop outs not considered	Unclear (matching approach not reported)
Gunter et al (2018) (pilot 2) [51]	Yes	No- uncontrolled	Yes	Yes	N/A- event rates	Yes	Yes	No- impact of drop outs not considered	Yes
Gunter et al (2016) (pilot 1) [50]	Yes	No- uncontrolled	Yes	Yes	N/A- experience metrics	Yes	Yes	No drop outs	Yes
Kariyawasam et al (2017) [45]	Yes	Yes	Unclear- demographics for both groups not reported	Unclear- historic control	Yes	Yes	Yes	Unclear, results incomplete	Yes
Hartup et al (2022) [29,46]	Yes	Yes	Unclear- demographics for both groups not reported	Yes	Yes	Yes	Yes	No- impact of drop outs not considered	Unclear, results incomplete
Duettmann et al (2021) [34]	Yes	No- uncontrolled	Yes	Yes	Unclear, results incomplete	Unclear, results incomplete	Unclear, results incomplete	Unclear, results incomplete	Unclear, results incomplete
Baig et al (2015) (pilot 1) [56]	Yes	No- uncontrolled	Yes	Yes	N/A- experience metrics	Yes	Yes	No drop outs	Yes
Baig et al (2014) (pilot 2) [44]	Yes	No- uncontrolled	Yes	Yes	N/A- validation metrics	Yes	Yes	No drop outs	Yes
