# Supplementary material for: Co-Designing Remote Patient Monitoring Technologies for Inpatients: Systematic Review
Source: J Med Internet Res. 2024 Oct 15;26:e58144. doi: 10.2196/58144 (PMC11522647; doi:10.2196/58144)
Supplement: Multimedia Appendix 4 [file jmir_v26i1e58144_app4.pdf]

Study ID	Domain									
	1. Congruity between philosophical perspective and the research methodology	2. Congruity between research methodology and the research question	3. Congruity between the research methodology and data collection methods	4. Congruity between the research methodology and data analysis	5. Congruity between the research methodology and the interpretation of results	6. Statement locating the researcher culturally or theoretically	7. Influence of the researcher on the research, and vice- versa, addressed	8. Participants, and their voices, adequately represented	9. Ethical approval obtained	10. Relationship between the findings reported and conclusions made
Naeemabadi et al (2020) Iteration 4 and (pilot 1) [48]	Yes	Yes	Yes	Yes	Yes	No, not reported	No, not reported	No, lack of supporting quotes	Yes	Yes
Naeemabadi et al (2020) Interaction 5 (pilot 2) [48]	Yes	Yes	Yes	Yes	Yes	No, not reported	No, not reported	Yes	Yes	Yes
Vaughn et al (2020) [40]	Yes	Yes	Unclear-semi-structured interviews but no audio recording	Yes	Yes	No, not reported	No, not reported	No, lack of supporting quotes	Yes	Yes
Blair et al (2022) [41]	Yes	Yes	Yes	Yes	Yes	No, not reported	Yes	Yes	No, not reported	Yes
Nielsen et al (2020) [53]	Yes	Yes	Yes	Yes	Yes	Yes	Yes, partially	Yes	Yes	Yes
An Qingfan et al (2021) (Aim 2) [38]	Yes	Yes	Yes	Unclear	Yes	No, not reported	No, not reported	Unclear, limited data presented	Yes	Yes
