# Supplementary material for: Co-Designing Remote Patient Monitoring Technologies for Inpatients: Systematic Review
Source: J Med Internet Res. 2024 Oct 15;26:e58144. doi: 10.2196/58144 (PMC11522647; doi:10.2196/58144)
Supplement: Multimedia Appendix 5 [file jmir_v26i1e58144_app5.pdf]

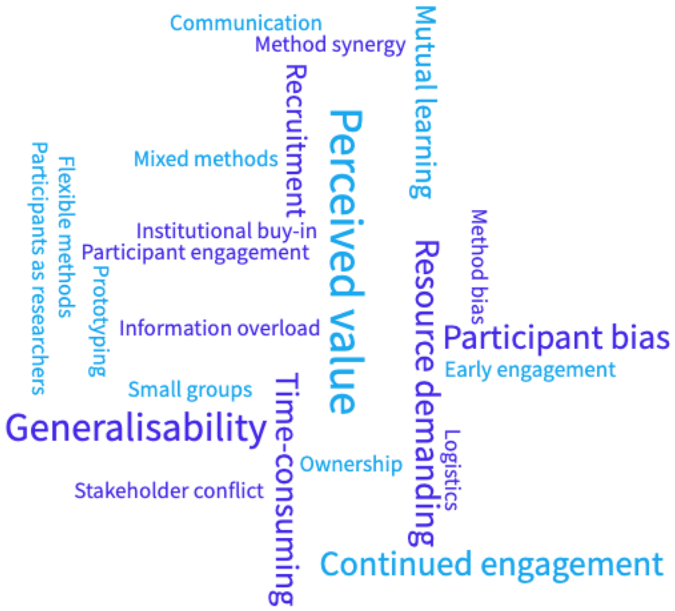

Word cloud depicting frequency of reported barriers (dark blue) and enablers (light blue) to the co-design process (larger words represent higher frequency of reporting).
